# Motor system contributions to verbal and non-verbal working memory

**DOI:** 10.3389/fnhum.2014.00753

**Published:** 2014-09-24

**Authors:** Diana A. Liao, Sharif I. Kronemer, Jeffrey M. Yau, John E. Desmond, Cherie L. Marvel

**Affiliations:** ^1^Department of Neurology, Johns Hopkins University School of MedicineBaltimore, MD, USA; ^2^Neuroscience Institute, Princeton UniversityPrinceton, NJ, USA; ^3^Department of Neuroscience, Baylor College of MedicineHouston, TX, USA; ^4^Department of Psychiatry and Behavioral Sciences, Johns Hopkins University School of MedicineBaltimore, MD, USA

**Keywords:** working memory, TMS, motor system, motor cortex stimulation, visual cortex, Sternberg memory task, verbal working memory, non-verbal working memory

## Abstract

Working memory (WM) involves the ability to maintain and manipulate information held in mind. Neuroimaging studies have shown that secondary motor areas activate during WM for verbal content (e.g., words or letters), in the absence of primary motor area activation. This activation pattern may reflect an inner speech mechanism supporting online phonological rehearsal. Here, we examined the causal relationship between motor system activity and WM processing by using transcranial magnetic stimulation (TMS) to manipulate motor system activity during WM rehearsal. We tested WM performance for verbalizable (words and pseudowords) and non-verbalizable (Chinese characters) visual information. We predicted that disruption of motor circuits would specifically affect WM processing of verbalizable information. We found that TMS targeting motor cortex slowed response times (RTs) on verbal WM trials with high (pseudoword) vs. low (real word) phonological load. However, non-verbal WM trials were also significantly slowed with motor TMS. WM performance was unaffected by sham stimulation or TMS over visual cortex (VC). Self-reported use of motor strategy predicted the degree of motor stimulation disruption on WM performance. These results provide evidence of the motor system’s contributions to verbal and non-verbal WM processing. We speculate that the motor system supports WM by creating motor traces consistent with the type of information being rehearsed during maintenance.

## Introduction

Working memory (WM) represents the ability to temporarily store or manipulate information that is briefly held in mind (Baddeley, 1992). WM is important for performing daily activities, such as cooking, driving, conversing, writing, navigating, and problem solving. A popular model of WM, developed by Baddeley and Hitch (1974) proposes that WM is defined by three primary components: (1) a central executive that works as a supervisory system to allocate attentional resources, and two storage systems; (2) the phonological loop that manages verbalizable content; and (3) the visuo-spatial sketchpad that manages visual (and often non-verbalizable) content (Baddeley, 2003).

The phonological loop can be further divided into two subcomponents: the phonological store, which passively holds memory traces for several seconds, and an articulatory control process, which refreshes the memory trace via active rehearsal. Active rehearsal may involve an inner speech mechanism in which people “talk” covertly in the absence of overt speech production (Baddeley, 1992; Marvel and Desmond, 2010, 2012; Koziol et al., 2014). Neuroimaging studies of verbal WM suggest localization of the phonological store resides in the inferior parietal lobule/supramarginal gyrus, and articulatory control in the left premotor frontal regions, Broca’s area, the supplementary motor area (SMA) and cerebellum (Paulesu et al., 1993; Chein and Fiez, 2001; Chen and Desmond, 2005a). It is thought that this brain activity, comprised mainly of secondary motor-related structures, represents an inner speech process that supports active phonological rehearsal (Ackermann et al., 2004, 2007; Marvel and Desmond, 2012; Ackermann, 2013; Koziol et al., 2014). Secondary motor-related activity has also been observed in neuroimaging studies of mentally simulated movement (Guillot et al., 2009; Hétu et al., 2013; Langner et al., 2014), suggesting that the motor system may broadly support WM by creating internal motor traces that are consistent with an action associated with the information held in mind.

Verbal WM is often studied in the laboratory using the Sternberg task (Sternberg, 1966). The Sternberg task consists of three phrases: (1) an encoding phase where visually presented information is converted to a mental representation; (2) a maintenance phase, in which this information is rehearsed across a brief delay; and (3) a retrieval phase during which a probe is presented and compared to the encoded information. Methodologically, the Sternberg task is advantageous because the each phase can be isolated and examined independently. Moreover, the Sternberg task easily generalizes to WM memory functions of daily life (e.g., reading and rehearsing a phone number before dialing it). The Sternberg task has been successfully combined with neuroimaging. fMRI studies have shown that secondary motor areas are most active during the encoding and maintenance phases, and this activity scales with WM load (Chein and Fiez, 2001; Chen and Desmond, 2005b; Kirschen et al., 2005; Marvel and Desmond, 2010, 2012).

Neuroimaging is a successful tool in cognitive neuroscience, yet its measures reflect correlation rather than causation. Meanwhile, neuropsychological approaches that link specific behavioral impairments to areas of brain damage can infer causation but only if lesions are circumscribed to a particular brain region. Lesion-based studies have suggested that damage to the motor system can impair WM (Malouin et al., 2004, 2012; Ravizza et al., 2006; Ziemus et al., 2007; Kirschen et al., 2008). However, lesions in these studies varied in terms of size and location, and potentially affected both motor and cognitive systems, making it difficult to determine causality. Moreover, across lesion studies, there is often variability of test protocols and in time lapse between neurological insult and assessment, which can also make it difficult to generalize results for interpretation. Such limitations can be addressed using non-invasive brain stimulation techniques, such as transcranial magnetic stimulation (TMS), to create a “virtual lesion” that temporarily disrupts neuronal activity in healthy adults (Pascual-Leone et al., 2000). Accordingly, TMS can provide evidence of a causal link between stimulation site and task performance.

TMS applied to the primary motor cortex (M1) elicits activity in additional nodes of the motor system, including the premotor cortex, SMA, and cerebellum. This has been demonstrated in studies using paired-pulse TMS and concurrent TMS-fMRI (Bestmann et al., 2004; Yau et al., 2013). Thus, multiple nodes within the motor system can be disrupted through the application of TMS to M1. A practical consideration in accessing the motor system this way is that when suprathreshold TMS is applied to hand region of M1, this creates an observable muscle response in the contralateral hand, thereby confirming accuracy of the stimulation site.

The current study combined TMS with the Sternberg task in order to examine the causal relationship between the motor system and WM. Stimulation was delivered over the hand region of the left primary motor cortex (M1) in order to access the secondary motor system. We predicted that TMS applied to M1 during the maintenance phase of the Sternberg paradigm would disrupt verbal WM rehearsal mechanisms. Additionally, the magnitude of disruption would scale with load: stimuli that required heavier phonological demands during WM would be more disrupted by motor stimulation.

## Materials and methods

### Participants

Twenty-seven healthy, right-handed, subjects (mean age = 21.19 ± 2.02 years, 13 females) were recruited. All participants were native English speakers and unable to read, write, or speak Chinese. None had a history of psychiatric or neurologic conditions, head injury resulting in loss of consciousness for more than 5 min, or were currently taking medications known to influence cognition. Written informed consent was obtained prior to participation, and participants were financially compensated for their time. This study was approved by the Johns Hopkins University School of Medicine Institutional Review Board.

### Experimental design

A within-subject design protocol was administered with a modified Sternberg paradigm (Sternberg, 1966) of three blocks of 48 trials for each participant. Each block of trials was tested under one of three TMS site applications: (1) left primary motor cortex (M1); (2) visual cortex (VC); and (3) a sham condition (Sham) in a counter-balanced order. Each block included three stimulus trial types: (1) real words; (2) pseudowords; and (3) Chinese characters. We predicted that the phonological WM load would be dissimilar across stimulus types. Pseudowords would heavily rely on inner speech and the phonological loop, representing a high WM load. By contrast, real words would be familiar, associated with semantic content, requiring limited WM load. Finally, Chinese characters were included as a non-verbalizable (and non-phonological) control condition to our participants who could not speak or read Chinese.

**Figure 1:**
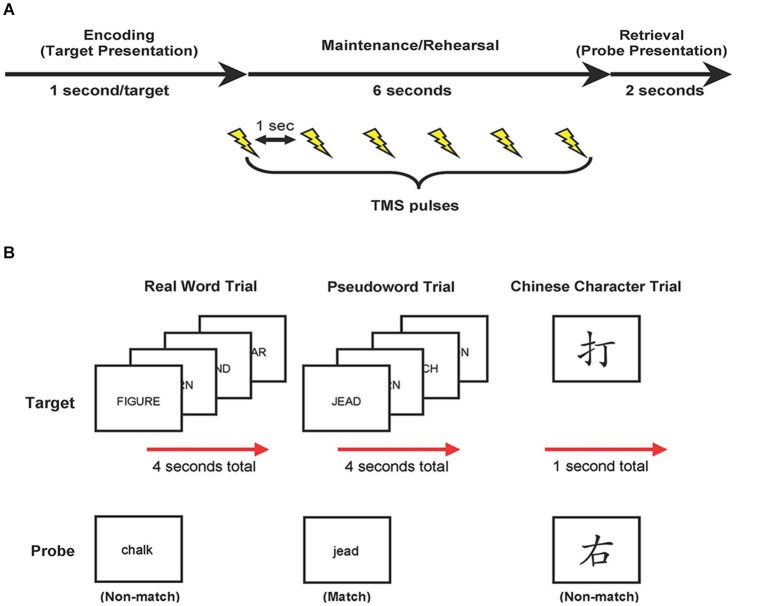
**Study design. (A)** Trial events. Target items were presented, followed by a 6-s delay. During this delay, target items were rehearsed while TMS was applied (6 pulses each separated by 1 s). At the end of each trial, participants compared a probe item to the targets and indicated match or non-match with a button press response. **(B)** Examples of real word, pseudoword, and Chinese character trial types.

Each trial began with visual presentation of the stimuli. Four real words or pseudowords were presented, in upper-case letters sequentially for 1 s each, or a single Chinese character was presented for 1 s (encoding phase; see Figure 1). Targets were replaced with a blank screen, and participants were required to covertly rehearse these targets for 6 s (maintenance phase). No further instructions were given regarding rehearsal strategy. During the maintenance phase, six TMS pulses (1-s inter-pulse interval) were applied. Finally, a probe item appeared, in lower-case, on the screen for up to 2 s, and participants determined if the probe matched one of the previously presented targets (retrieval phase). Participants responded via button-press with their left hand (ipsilateral to that M1 stimulation site) using a serial response box (Psychology Software Tools, Inc., Sharpsburg, PA). Participants pressed their middle finger to indicate a match, and their index finger to indicate a non-match, and were required to respond while the probe item was displayed (i.e., maximum of 2 s). The probe item disappeared upon response or after 2 s. The inter-trial interval was 3 s. The total duration for each block of 48 trials was 10 min and 24 s, with a rest period of 10 min between blocks. Trial types and probe types (i.e., match and non-match) were pseudo-randomized with no more than three of the same type presented consecutively. Half of all trials included a matching probe type. Stimuli were presented using Eprime 2.0 (Psychology Software Tools, Inc., Sharpsburg, PA).

Participants were asked to avoid overt talking or body movements during rehearsal and to respond as quickly and accurately as possible. Participants were given 12 practice trials of the WM paradigm prior to beginning the official test.

The dependent measures were response time (RT) and accuracy. RT duration was measured between onset of the probe and the button-press response. Inaccurate response trials were disregarded from RT analyses. Accuracy scores were defined as the percentage of trials answered correctly. Mean RTs and accuracy scores were calculated for each block at the three stimulation sites (left M1, VC, and Sham) and the three trial types (real words, pseudowords, Chinese characters). For each trial type, each subject’s mean RT from the Sham block was subtracted from the mean RTs on M1 and VC blocks. Doing so canceled out the potential effects of TMS coil itself, including acoustics and startle response (Xu-Wilson et al., 2011). Thus, the RT (or accuracy) difference between M1—Sham and VC—Sham represented the TMS effect on M1 and VC RT (or accuracy) by trial type for each subject. A 2 (TMS effect of stimulation site: M1 and VC) × 3 (trial type: real words, pseudowords, and Chinese characters) repeated-measures analysis of variance (rmANOVA) was conducted on RT and accuracy data. All statistics were conducted using SPSS (IBM SPSS Statistics version 22.0) and Matlab scripts (Version 7.11.0 (R2010b), Natick, Massachusetts: The Mathworks Inc., 2012).

### Stimuli characteristics

A total of 216 unique real words were presented as targets (192) or probes (24 non-match probe types), ranging from 75–10,595 per million in frequency, with a mean of 261.54 per million and standard deviation of 602.05 (Kucera and Francis, 1967). All words contained 4–7 letters with a mean of 5.34 letters (+/−1.08), and 1–3 syllables with a mean of 1.55 syllables (+/−0.58). Their mean orthographic neighborhood density was 4.69 (+/−5.13), ranging from 0–26, and their mean phonologic neighborhood density was 11.27 (+/−10.87), ranging from 0–40 (Balota et al., 2007). Within a trial, exactly two phonemes were shared across all four targets. Real word targets and the respective probe within a trial did not fall within the same semantic category (Battig and Montague, 1969). Similarly, 216 pseudowords were derived from real words by changing 1 or 2 phonemes in either the first or second half of the word, with the length and the number of syllables conserved. This conversion process was controlled. Half of the targets in each pseudoword trial had the first half of the corresponding word changed and half of the targets had the second half of the word changed (e.g., “Bottle” > “Tottle” and “System” > “Systeg”).

A total of 72 unique Chinese characters were presented as targets (48) or probes (24 non-match probe types). Chinese characters were matched for visual similarity. All Chinese characters were composed of 5–6 strokes and the total visual space occupied by each character (i.e., ratio of black to white pixels) was equated. Only a single Chinese character was shown as a target during encoding. During pilot testing, a single target character yielded comparable accuracy with that of the four-item real word and pseudoword trials, whereas accuracy dropped with two or more target Chinese characters.

### TMS parameters

TMS was performed with a biphasic Magstim Rapid stimulator and a 70 mm figure-of-eight coil (The Magstim Company Ltd.). Coil position relative to the participant’s scalp was monitored using Brainsight (Rogue Research; Montreal Quebec, Canada). Coil movement during the experiments was minimal (<1 cm displacement from the target sites).

To determine each participant’s resting motor threshold (RMT), the coil was placed tangentially to the skull with the handle at a 45° angle to the anterior-posterior axis. Single TMS pulses were applied to the participant’s left M1 to locate the site where TMS pulses elicited the largest twitch in the first dorsal interosseous muscle of the right hand. This location was used to establish RMT and set as the motor target site. RMT was determined as the minimum stimulator intensity that elicited a visible muscle twitch in ≥ 5 of 10 consecutive TMS pulses (Rossini et al., 1994). TMS intensity during the experiment was set to 90% RMT. All stimulation parameters were in accordance with the safety guidelines for TMS (Wassermann, 1998).

Two additional stimulation sites were determined. In the Sham condition, the coil was positioned over the vertex (location Cz of the EEG 10–20 system), defined as the intersection between the left-right tragus and nasion-inion axis (Teplan, 2002). Above the vertex, the coil was flipped 90° perpendicularly on top of the head so that the outer edge of the coil made contact with the scalp. During the Sham condition participants would have similar auditory experiences to that of the M1 stimulation condition (i.e., hearing the clicks of the coil). We additionally included a comparative stimulation condition in which the coil was placed over the VC. To stimulate the VC, the coil was placed on the occipital midline, 4 cm above the inion, in close proximity to the Oz site, with the handle pointed superiorly. Stimulation to the VC was not expected to interfere directly with the inner speech motor pathway, but it was possible that VC stimulation would disturb a visual imagery strategy for WM rehearsal. Thus, data for M1 and VC stimulation conditions were compared directly, subtracting out the Sham condition from each.

### Post-experimental testing

Upon completing the modified Sternberg task, participants responded to a questionnaire. The objective was to explore the use of motor rehearsal strategies (e.g., subvocal rehearsal, mental tracing) vs. a visual imagery rehearsal strategies (e.g., visual snapshot). Participants were asked to rate their subjective use of a “motor” or “visual” strategy for each trial type. These measures were rated using a modified Likert scale from 1 (lowest) to 7 (highest). No further instructions or examples were provided regarding the definition of strategy types, and therefore, a subject’s definition of “motor” and “visual” strategies were subjective. Then participants described the specific strategy that they used in an open-ended response section probing, “What was your strategy for rehearsing real words? Pseudowords? Chinese characters?” Open-ended responses were used to explore the specifics of rehearsal strategies and were not quantified for statistical analyses. Because this questionnaire was added to the study while it was in progress, only 22 of 27 participants completed the questionnaires.

## Results

### Working memory task

**Figure 2:**
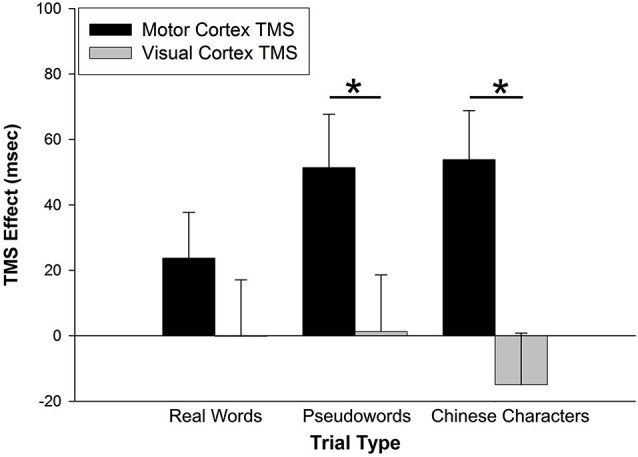
**TMS effects on RT**. The TMS effect represents the difference between primary motor cortex (M1) and visual cortex (VC) RT minus Sham RT. M1 stimulation slowed RTs for pseudoword and Chinese character trial types. VC stimulation had no effect on RTs. Error bars denote standard error. * *p* < 0.01.

The prediction that M1 stimulation would disproportionately affect performance for pseudowords trials was tested through an interaction analysis of trial type by stimulation site. For RT data, a 2 (TMS effect on stimulation site) × 3 (trial type) repeated-measures analysis of variance (rmANOVA) yielded a main effect of stimulation site, *F*_(1,26)_ = 15.023, *p* = 0.001 (Figure 2). There was no main effect of trial type, *F*_(2,52)_ = 0.488, *p* = 0.617. There was, however, an interaction of stimulation site by trial type, *F*_(2,52)_ = 4.040, *p* = 0.023. *Post-hoc* paired-sample *t*-tests (Bonferroni corrected) indicated that the TMS effect was greater for M1 than VC for pseudoword trials (*t*_(26)_ = 3.000, *p* = 0.006), but not for real word trials (*t*_(26)_ = 1.731, *p* = 0.095). Counter to our hypothesis, the TMS effect also was greater for M1 than VC for Chinese character trials (*t*_(26)_ = 4.448, *p* < 0.001).

**Figure 3:**
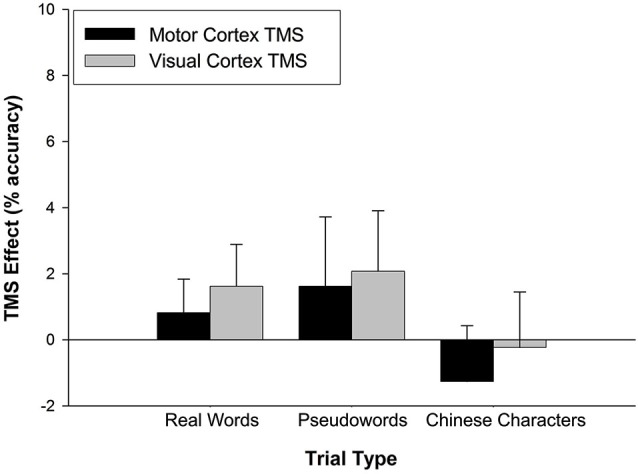
**TMS effects on accuracy**. The TMS effect represents the difference between M1 and VC accuracy minus Sham accuracy. Stimulation site had no effect on accuracy across trial types. Error bars denote standard error.

Similar analyses were applied to accuracy performance. A 2 (TMS effect on stimulation site) × 3 (trial type) rmANOVA was conducted but neither main nor interaction effects achieved statistical significance (all *p* > 0.5) (Figure 3).

### Effect of M1 stimulation on motor slowing

**Figure 4:**
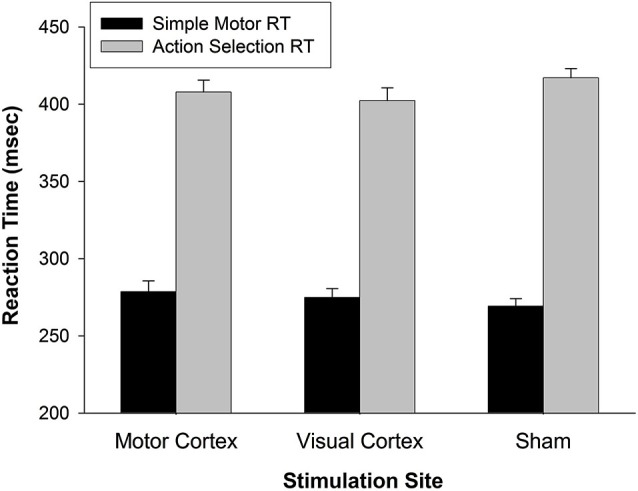
**General effect of TMS on reaction time**. In a simple motor response task, RTs for motor TMS were comparable to RTs for visual TMS and Sham. When a simple action selection was introduced (choose button 1 or 2), RTs slowed generally (*p* < 0.001), but not as a function of stimulation site (*p* < 0.14). These findings indicate that motor TMS effects on finger response were not the primary cause of slowed RTs during working memory (in Figure 2). Error bars denote standard error.

The effect of M1 stimulation on RT was greater than that of Sham (relative to a value of 0 on the *y*-axis in Figure 2) for pseudoword trials (*t*_(26)_ = 3.159, *p* = 0.004) and Chinese character trials (*t*_(26)_ = 3.593, *p* = 0.001). Even though it was not significant for real word trials (*t*_(26)_ = 1.693, *p* = 0.102), the RT difference skewed toward slowing with M1 stimulation. To rule out the possibility that M1 stimulation slowed general sensorimotor processing speed (an effect that may have been exacerbated by cognitive load), we tested five additional participants on two separate tasks with TMS. One task assessed simple reaction time by asking participants to press a button as soon as they saw a plus sign appear on the screen (shown for 1000 ms, for 32 trials, inter-trial interval = 1400–2000 ms) with the index finger of their left hand. A second task increased difficulty by introducing a decision component to the motor act (i.e., action selection): Participants were presented with a “1” or “2” (shown for 1000 ms, for 32 trials, inter-trial interval = 1400–2000 ms) and pressed the left or right button, respectively, with their left hand. All subjects were right-handed. Participants performed each task three times—while receiving TMS to left M1, VC, or sham sites. The order of stimulation site was counterbalanced across participants. A 2 (task: motor response vs. action selection) × 3 (stimulation site) rmANOVA indicated that responses were significantly slower for the action selection than motor response task (*F*_(1,4)_ = 489.861, *p* < 0.001) (Figure 4). There was, however, no main effect of stimulation site (*F*_(2,8)_ = 0.049, *p* = 0.952) nor interaction of task × stimulation site (*F*_(2,8)_ = 2.589, *p* = 0.136). Thus, M1 stimulation did not appear to slow RT *per se* during a simple motor response task and did not exacerbate motor slowing with cognitive load.

### Relation between performance and strategy

**Figure 5:**
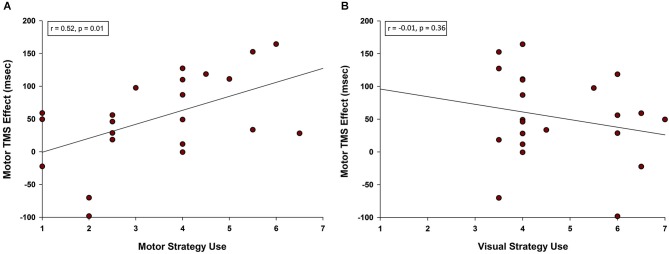
**Correlation between motor TMS effects and use of motor strategy. (A)** A positive correlation was observed between self-reported use of motor rehearsal strategy and the motor TMS effect. Participants who were more likely to rely on a motor strategy when rehearsing pseudowords and Chinese characters were more disrupted by M1 stimulation. **(B)** There was no correlation between self-reported use of visual rehearsal strategy and the motor TMS effect.

Given that primary motor cortex stimulation affected RT for pseudoword and Chinese character trials specifically, we were interested to know whether self-reported strategies predicted TMS effects on these two trial types. Post-experimental questionnaires indicated that rehearsal of stimuli involved both motor and visual strategies. Ratings scaled from 1 (low) to 7 (high) and were not exclusive. That is, it was possible for a participant to report high use of both visual and motor strategies for the same trial type. Examples of motor strategies included mentally tracing, writing, or drawing (Chinese characters) or subvocally repeating (pseudowords). Examples of visual strategies included holding a “snapshot” of the image in mind and turning the characters into familiar real-world objects. We averaged the motor strategy ratings and M1 TMS effects on RT for pseudoword and Chinese character trial types. A Pearson product-moment correlation coefficient between motor strategy ratings and the M1 TMS effect revealed a positive correlation (*r* = 0.521, *n* = 22, *p* = 0.013; see Figure 5A). Thus, the more strongly a participant relied on motor rehearsal strategies, the greater the RT disruption associated with motor stimulation. This relationship was selective: there was no correlation between the M1 TMS effect and visual strategy ratings (*r* = −0.207, *n* = 22, *p* = 0.356, Figure 5B).

## Discussion

The current investigation examined the role of the motor system in WM by stimulating motor cortex during silent rehearsal of verbal and non-verbal content. By stimulating the primary motor cortex, we also stimulated secondary motor regions (Lefaucheur et al., 2014). We hypothesized that motor cortex stimulation would disrupt verbal WM rehearsal mechanisms that rely on secondary motor regions and, therefore, disproportionately affect stimuli with the highest phonological load (i.e., pseudowords). Because unphonetic Chinese characters (to naïve English speakers) discouraged verbalizable rehearsal strategies, we anticipated that their rehearsal would not be affected by motor stimulation. Our findings supported and expanded these hypotheses.

Consistent with our hypothesis, TMS targeting motor cortex slowed RT for pseudowords. Surprisingly, motor cortex stimulation also slowed RT for Chinese characters. This result could not be explained simply as TMS-induced slowing of button presses, as a secondary analysis demonstrated that RTs for button pressing, *per se*, were unaffected by the M1 stimulation. Moreover, self-reported motor strategies predicted the degree of motor stimulation disruption on RTs. These results indicate that motor activity contributed selectively to WM performance. Neuroimaging studies have shown that WM rehearsal of verbal content involves non-primary motor areas consisting of the left premotor cortex, SMA, and superior cerebellum (Paulesu et al., 1993; Chein and Fiez, 2001; Chen and Desmond, 2005a; Marvel and Desmond, 2010, 2012). However, non-verbal WM rehearsal can also involve the premotor cortex (left and right), SMA, and superior cerebellum (Champod and Petrides, 2007). Our intention was to disrupt motor-based strategies during phonological rehearsal, yet primary motor cortex stimulation appears to have interfered with motor-based strategies for non-verbal WM rehearsal too.

Although we delivered TMS directly to the primary motor cortex, stimulation likely affected both local processing in the targeted area and remote processing in distributed, interconnected motor regions. Indeed, the distant actions of TMS applied to the motor system have been demonstrated consistently in physiology and imaging studies (Lefaucheur et al., 2014). Concurrent TMS-fMRI studies have shown that stimulation targeting the primary motor cortex evoked BOLD signal changes in connected cortical and subcortical motor-related brain regions including the premotor cortex, SMA, primary and secondary somatosensory cortex, cerebellum, and basal ganglia (Bestmann et al., 2003; Denslow et al., 2005; Yau et al., 2013). Notably, studies using TMS at intensities below RMT, as with the levels used in this study, have evoked clear responses in secondary motor areas (Bestmann et al., 2004; Hanakawa et al., 2009). Functional coupling within the motor network has also been revealed by paired-pulse TMS experiments, demonstrating that TMS targeting a host of secondary motor structures, including premotor cortex, SMA, and cerebellum, influences motor cortex responses (Reis et al., 2008). Thus, while we cannot definitively claim that the WM disruptions observed here resulted from distant effects of primary motor cortex TMS, the strong functional connectivity within the distributed motor system makes this an attractive claim to test explicitly in future TMS studies.

Participants could not verbalize Chinese characters explicitly to exploit an inner-speech rehearsal mechanism. Nonetheless, self-reports revealed that participants often rehearsed Chinese character stimuli using an alternative motor-based strategy that involved mental drawing or tracing. These subjective reports and our result patterns are consistent with the notion that internal motor traces are created to represent WM contents and to strengthen WM capacity (Ackermann et al., 2004; Ravizza et al., 2006; Marvel and Desmond, 2012; Koziol et al., 2014). Because participants were silent and still during rehearsal, motor sequence generation for WM maintenance appears to occur in the absence of intention or execution of movement. While the relationship between different motor rehearsal mechanisms remains to be established, our results clearly imply that the motor system is recruited for verbal and non-verbal WM.

A similar process is used to mentally represent observed movements. Using fMRI, Langner et al. (2014) visually presented motor sequences to participants at the start of each trial. Motor sequences were reproduced by the participant after a delay. The authors reported that motor-related activity (basal ganglia, premotor cortex, SMA, and cerebellum) overlapped during sequence encoding and recall. They interpreted their results as “encoding to-be-reproduced visuo-spatial sequences may initially entail imitation-related, concrete motor representations that, in turn, are retained in a more abstract form” (p. 10). This process, they posited, represented an execution-related WM subsystem that facilitates memory for sensory input based on how the information will be used. Our findings extend those of Langner et al. by demonstrating motor involvement for sensory input (e.g., pseudowords and Chinese characters) without a requirement to physically reproduce the information. These mechanisms may be similar to the way in which mental representations of actions are generated during action observations, such as activity in the hand premotor cortex area when observing hand movements (Buccino et al., 2001). In fact, even simply reading action phrases leads to activity in the specific brain region the associated body part specifically, as has been demonstrated for the hand, mouth, and foot premotor regions (Aziz-Zadeh et al., 2006). Thus, we speculate that, just as the motor system is activated somatotopically when observing or imagining actions, it is also possible that motor regions are activated somatopically when rehearsing information over brief delays. Motor traces may be created within brain regions that would be used to speak, draw or otherwise reproduce the information, even without an intention to execute. Motor traces may create redundancy with other types of representations, such as visual or acoustic, and thereby reinforces the memory of the information held in mind. Motor representations are more likely to activate during the rehearsal process as WM load increases (Marvel and Desmond, 2012).

The results of this study underscore a role for the motor system in WM. However, the reverse relation may also be true. Wilson and Fox (2007) demonstrated that gesturing was susceptible to WM biases observed for verbal content, including the similarity effect (i.e., lists of similar words or gestures are difficult to remember), suppression effect (i.e., repetitive mouth movements or gestures during rehearsal disrupt language or gesture recall, respectively), and length effect (e.g., list of long words or gestures are more difficult to recall than short). Likewise, American Sign Language (ASL) speakers present similar WM biases as English speakers (Wilson and Emmorey, 2006). These findings demonstrate that verbal and non-verbal WM draw upon overlapping systems, particularly if the non-verbal stimuli can be represented visuospatially. Stimuli that are easily deconstructed into individual and continuous components (e.g., lines, shapes, and patterns) may be conducive to mental tracing strategies, such as those reported for the rehearsal of Chinese characters in this study.

It is worth noting that the site of left motor cortex stimulation was targeted over the hand region. Studies have indicated a link between the left motor hand area and language (e.g., silent word reading) (Meister et al., 2003, 2009). Thus, targeting the left motor hand area may have interfered with verbal WM directly. Targeting the left motor hand area also may have interfered with mental drawing and tracing for these right-handed participants. If so, disruption of the left motor hand area may have been the primary (or even exclusive) source of our observed effects on rehearsal for pseudowords and Chinese characters. A future direction of research could explore the specificity of TMS effects on WM, comparing effects of stimulation on the hand region vs. mouth and foot regions, as well as secondary motor areas, such as the lateral cerebellum, SMA, and premotor cortex.

In summary, findings from this study demonstrated specificity of motor network involvement in the rehearsal of unfamiliar, phonologically demanding verbal content as well as for non-verbal content. The role of the motor system seems particularly important when the information to be held in mind would benefit from a covert motor strategy, such as inner speech or mental drawing. These results have implications for any clinical disorder that involves motor deficits, as damage to primary or secondary motor brain regions may impact WM function.

## Conflict of interest statement

The authors declare that the research was conducted in the absence of any commercial or financial relationships that could be construed as a potential conflict of interest.
